# Predictors of Antenatal Care Service Utilization Among Women of Reproductive Age in Ethiopia: A Systematic Review and Meta-Analysis

**DOI:** 10.3390/jcm14072517

**Published:** 2025-04-07

**Authors:** Amanuel Yoseph, G. Mutwiri, Francisco Guillen-Grima

**Affiliations:** 1 School of Public Health, College of Medicine and Health Sciences, Hawassa University, P.O. Box 1560 Hawassa, Ethiopia; 2 School of Public Health, University of Saskatchewan, Saskatoon, SK S7N 5B5, Canada; george.mutwiri@usask.ca; 3 Department of Health Sciences, Public University of Navarra, 31008 Pamplona, Spain; 4 Group of Clinical Epidemiology, Area of Epidemiology and Public Health, Healthcare Research Institute of Navarre (IdiSNA), 31008 Pamplona, Spain; 5 CIBER in Epidemiology and Public Health (CIBERESP), Institute of Health Carlos III, 46980 Madrid, Spain; 6 Department of Preventive Medicine, Clinica Universidad de Navarra, 31008 Pamplona, Spain

**Keywords:** antenatal care, utilization, predictors, socioeconomic status, women, reproductive age groups, planned pregnancy, education status, mass media exposure, Ethiopia

## Abstract

**Objective:** This study aimed to provide pooled predictors of ANC (antenatal care) service use among women of reproductive age in Ethiopia. **Methods:** Studies were systematically searched using PubMed, Medline, CINAHL, EMBASE, and Google Scholar. The Newcastle–Ottawa scale (NOS) tool was utilized for quality assessment (risk of bias). All data analyses were conducted by utilizing Stata version 17. A random-effects model was used to obtain the pooled predictors of ANC use. The publication bias was checked using a funnel plot and Egger’s regression test. **Results:** Twenty-two studies with a sample size of 25,671 were included in this review. The identified predictors of ANC service use were highest wealth rank (AOR 1.92 [95% CI: 1.53–2.31]), formal women education (AOR 2.40 [95% CI: 1.75–3.06]), formal husband education (AOR 1.49 [95% CI: 1.36–1.66]), women age ≥ 20 (AOR 1.75 [95% CI: 1.47–2.17]), mass media exposure (AOR 1.44 [95% CI: 1.21–1.66]), good maternal knowledge about the pregnancy complication (AOR 1.49 [95% CI: 1.11–1.88]), planned pregnancy (AOR 1.59 [95% CI: 1.28–1.91]), women autonomy (AOR 1.42 [95% CI: 1.23–1.62]), and positive husband attitude about the ANC service use (AOR 2.63 [95% CI: 1.47–3.79]). **Conclusions:** Several predictors have increased the ANC service utilization, like wealth status, women’s and their husbands’ education, older/increasing women’s age, media exposure, maternal knowledge about pregnancy complications, planned pregnancy, women’s autonomy to decide on household health care, and positive husband attitude about the ANC service utilization.

## 1. Introduction

Though pregnancy has been taken as an achievable natural experience that is emotional or sensitive to women, many women pass through several problems where they suffer and even die [1,2,3,4]. Due to this, there is a demand to invest or advance in the health care of mothers. Investment can be accomplished through different techniques; one is promoting the use of ANC services [4,5,6,7].

ANC service utilization remains essential for enhancing maternal and neonatal health outcomes [5,8]. It gives a chance for the pregnant mothers and health care providers to discuss adequate nutrition, identify obstetrics danger signs (ODS), and a childbirth plan [9,10,11], and protective care, such as the supply of ferrous sulfate or folic acid pills and tetanus toxoid immunization [4,10]. Moreover, a more recent study indicated that early childhood health practices gained during the prenatal period could provide context for the importance of proper antenatal care for long-term outcomes [12]. Due to the above benefits, at least eight ANC visits for mothers with a normal pregnancy have been recommended by the World Health Organization (WHO) to offer users a more women-centered and positive experience [13].

Globally, ANC service utilization was 72.9%, ranging from 53.3% in developing countries to 93.3% in developed countries [11]. Likewise, only 43% of women in Ethiopia benefited from four or more ANC visits during pregnancy [14]. Moreover, the coverage of ANC utilization varies by region, sub-region, and district in Ethiopia based on findings of studies and reports. The conclusions of these studies showed that the utilization of the ANC ranged from 12% to 94.8% [15,16,17,18,19,20,21,22,23,24,25,26,27,28,29,30,31,32,33,34]. The most recent study reported that at least one ANC visit among pregnant women was only 54.5% in Northern Ethiopia [35]. In addition, only 52% and 28.2% had one and four or more ANC visits per WHO recommendations in southern Ethiopia [36]. Furthermore, only 40% of pregnant women had four or more ANC visits, which ranged highest in Addis Ababa (70%) and least in the Afar region (3%) in Ethiopia based on recent study findings [37]. Similarly, only 23% of pregnant women attended at least four ANC visits in the Somali region [38]. The above variation in the coverage of ANC services is a significant barrier that influences Ethiopia’s progress toward achieving maternal and neonatal mortality statistics in line with the Sustainable Development Goal (SDG) 3 [6,8].

Based on the 2019 Mini Ethiopian Demographic and Health Survey (EDHS) report, the ANC service utilization increased from 28% in 2005 to 74% in 2019 [14]. However, there is little progress in closing the gap between regional states, and Ethiopia’s urban and rural differences persist [14,15,16,17,18,19,20,21,22,23,24,25,26,27,28,29,30,31,32,33,34]. In addition, approximately a third of mothers who accessed ANC services do not receive the whole package of services during their follow-up [14,39]. The several interconnected predictors have contributed to low ANC utilization. They can be categorized as socioeconomic and demographic, obstetric characteristics, health facility or organizational, service quality-linked predictors, proper infrastructure and health system, socio-cultural, and the lack of transport in rural settings [15,16,17,18,19,20,21,22,23,24,25,26,27,28,29,30,31,32,33,34]. Furthermore, the timely initiation of ANC services was impeded by socio-cultural misconceptions, such as the belief that pregnancy is just blood or water in the womb before four months of gestation [40].

However, previous studies reported controversial findings on the relationship between different predictors and ANC service utilization in various settings of Ethiopia [15,16,17,18,19,20,21,22,23,24,25,26,27,28,29,30,31,32,33,34]. For instance, some studies showed a positive association between women’s and husbands’ education and ANC utilization [24,26,28,30,33], while others showed no association [21,22,27]. Similarly, some studies reported the highest wealth rank and increased ANC utilization [26,30,33], whereas others showed no association [32]. Moreover, the prevailing evidence is conflicting between place of residence [15,17,20,26,27,33,34], women’s age [15,18,23,28,29,41], family income [15,16,17,18,19,21,22], women’s occupation [15,16,17,18,21,22,23,31,34], husband’s occupation [25,29,31], marital status [17,19,29,34], and women’s mass media exposure [16,17,20,23,26,42]. Keeping these controversies unanswered at the primary study level can lead to missing the target point of intervention strategies. Several small studies conducted on the predictors of ANC utilization in Ethiopia have been published, although the results vary from one place to another. This review and meta-analysis are thus required to provide a comprehensive picture of which determinants are essential and how much influence they have on ANC service utilization. These data are necessary for policymakers, implementers, and program administrators to detect gaps in the utilization of ANC and design comprehensive strategies and initiatives to boost service utilization. Furthermore, there is limited evidence on the predictors of ANC service utilization in Ethiopia. Therefore, we aimed to provide comprehensive evidence on pooled predictors of ANC service utilization among women of reproductive age in Ethiopia.

### Research Question

What are the pooled predictors of antenatal care service utilization among women of reproductive age in Ethiopia?

## 2. Materials and Methods

### 2.1. Study Design

A systematic review and meta-analysis were conducted to estimate ANC service use pooled predictors.

### 2.2. Eligibility Criteria to Include Studies

The following inclusion criteria comprised studies in this review and meta-analysis. Study design: all observational studies reported predictors of ANC service utilization after multivariable analysis in Ethiopia; study period: studies conducted from 2002 to 2022 were included. The starting period was considered 2002 because the Ethiopian government implemented focused ANC during this period; study setting: both community- and institution-based studies were considered; study subjects: women of reproductive age groups who were pregnant or gave birth at least once preceding the survey—exposure: determinants or predictors or associated factors of ANC service. The predictors are exposure or characteristics that influence the probability of ANC service utilization. These are socioeconomic and demographic, obstetric characteristics, health facility or organization, service quality-linked predictors, proper infrastructure, health system, and socio-cultural. Outcome: a woman with at least one ANC visit; language: A study containing full text only in English was included; publication status: unpublished and published studies.

### 2.3. Search Strategy

All studies were systematically searched from international databases utilizing keywords such as antenatal care, maternal health care, prenatal care, utilization, utilization, predictors, determinants, associated factors, women of reproductive age, and Ethiopia as search terms. PubMed, Medline, CINAHL, EMBASE, HINARI, the Cochrane Library, Google Scholar, and Google were searched to obtain studies carried out in Ethiopia between 2002 and 2022. This is the search strategy used for PubMed: (((((((((Antenatal care) OR (Maternal health care)) OR (Prenatal care)) AND (Utilization)) OR (Utilisation)) AND (Predictors)) OR (Determinants)) OR (Associated factors)) AND (Women of reproductive age)) AND (Ethiopia). The details of the search strategy for PubMed, Medline, CINAHL, and EMBASE are provided as Supporting Information (Supplementary File S1). The retrieved studies’ reference lists were reviewed to prevent the misuse of other relevant articles during the search process. We registered the systematic review in the system of PROSPERO prospective register with the registration number CRD42022322940. The review protocol was prepared and can be accessed from the PROSPERO register. The review protocol amendment was not made.

### 2.4. Method of Study Selection

We utilized the PRISMA flow chart to describe the flow of information via different stages of a systematic review [43]. We used four steps to assess the applicability of studies in our review based on the eligibility criteria. First, the duplicated studies were rejected from different sources. Second, the titles and abstracts were critically assessed based on the eligibility criteria. Articles that fulfilled the eligibility criteria in the second screening procedure were considered candidates for the third screening procedure. Third, each article’s full-text review was conducted based on eligibility criteria before being included. We contacted the corresponding author of studies when studies did not contain adequate data. We obtained clarification on the concerns and solved all doubts encountered. Finally, this review included twenty-two studies that fulfilled pre-determined inclusion criteria (Supplementary File S2). This review was prepared based on the PRISMA 2020 checklist, and the checklist is now provided as Supporting Information (Supplementary File S3).

### 2.5. Quality Assessment (Risk of Bias)

The review authors (AY and FGG) rigorously assessed all studies chosen to include in the review. The NOS tool was utilized to determine the methodological quality of observational studies in systematic reviews and meta-analyses and evaluate the risk of bias within involved studies [44]. The included studies were given a maximum of four stars in the subject selection, two stars in group comparability, and three stars in the outcome measurement procedure based on NOS. For observational studies, the methodological quality was categorized as low, moderate, or high when the NOS score fell between 0 and 4, 5–6, and >seven stars, respectively (see Supplementary File S4).

### 2.6. Data Abstraction or Extraction

Data abstraction format was used to extract data from included studies. Both authors constructed it with explicit exclusion and inclusion criteria. Both authors were involved in data abstraction format development to ensure that the tool accurately captured all needed data to respond to the review queries. Both authors conducted data abstraction from the included studies. From each included study, the authors collected the name of the first author(s), the proportion of ANC service use, predictors, response rate, study areas, participants, design, data collection period, years of publication, sample size, and AOR with 95% CI (Table 1). The abstracted data from the included studies are now provided as Supporting Information (Supplementary File S5).

### 2.7. Definition of Variables

ANC service utilization: Women with at least one ANC follow-up were utilizing the services.

Residence was divided into urban and rural. Rural residence was used as a reference category.

Age of the women: Women were allocated to age groups ≤19 and >19 years. The second group was utilized as the reference group.

Women/husband’s education status: (no formal education and have formal education). The first group was utilized as the reference.

Marital status was categorized as married and other groups (comprising single, widowed, and divorced). The other group was utilized as the reference group in our analysis.

Parity was grouped into women having 1–4 and more than four living children. The last one was utilized as a reference.

Category of pregnancy: it was assigned as planned vs. unplanned. The latter one was used as the reference point.

### 2.8. Statistical Data Analysis Methods

The data were extracted in Microsoft Excel format and exported to Stata version 17 software for analysis. We utilized figures and tables to summarize the descriptive results of the included studies. The I^2^ statistic was calculated to assess heterogeneity between studies, with a *p*-value < 0.05 indicating significant heterogeneity. If the I^2^ value is ≤50%, the studies are considered homogeneous, whereas a value >50% indicates high heterogeneity. In cases of high heterogeneity, a random-effects model is recommended for meta-analysis [45,46]. The random effect model was used due to heterogeneity in our review based on recommendations. Due to heterogeneity between studies, we implemented subgroup analysis based on the quality of studies, sample size, study design, year of data collection, and region. Also, a meta-regression model was utilized to detect the sources of variations based on the year of data collection, sample size, region, and design. We utilized a forest plot of AOR with a 95% Confidence Interval (CI) to measure pooled effect size estimates for the predictors of ANC service use. The sensitivity analysis evaluated the robustness or stability of the pooled estimates to the impact of individual studies and outliers. We utilized the funnel and counter-enhanced plot asymmetry and Egger’s test to check the presence of publication bias [47].

## 3. Results

### 3.1. Database Search Findings

A database search was conducted from 1 to 31 June 2023, and 711 studies were collected. Only 231 studies persisted following the rejection of duplicated records. We critically evaluated the titles and abstracts of the studies and rejected 169 studies based on the criteria. Only twenty-two studies remained following the application of the exclusion and inclusion criteria (see Supplementary File S6). We excluded forty studies after critically evaluating their full text from the review for several reasons (see Supplementary File S7).

### 3.2. Description of Included Study Features

We described the features of the included studies in a review (see Supplementary File S8). The majority, 20 (90.9%) of included studies, were cross-sectional, and only 2 employed a cohort design. The total sample size from twenty-two studies was 25,671 and ranged between 307 and 7908 women of reproductive age. The majority, 10 (45.46%) of studies, were from Oromia and South Nations Nationalities and Peoples Regional State (SNNPRS). Two regional and national-based studies were also included. Most study subjects were women of reproductive age who gave birth in the five years preceding the survey. The utilization of ANC service was wide-ranging, between 28.5% and 90.6%, in this review. Among twenty-two studies, eight were categorized as low-quality based on the NOS checklist evaluation (Supplementary File S4). The primary limitation of these low-quality primary studies was that they used inappropriate sampling techniques [15,19,21,31,42], the sample size calculation was not straightforward and acceptable [19], and the statistical data analysis procedure was not explained clearly [16,19,23,24,42].

### 3.3. Predictors of ANC Service Utilization

Of twelve studies that assessed the place of residence as a predictor for ANC service utilization, eight studies reported that urban residence increased the odds of ANC service utilization among Ethiopian women. However, the four studies revealed that urban residence had no significant association with ANC service utilization. The pooled data from this analysis reported that urban residence had no significant association with women’s ANC service utilization (AOR = 1.02; 95% CI: 0.82–1.21). The six studies evaluated the effect of wealth status on ANC service use. Five studies reported a significant positive association between the highest wealth rank and ANC service use among women. However, one study reported that wealth status did not affect ANC service utilization. The results from this analysis reported that the highest wealth rank increased the odds of ANC service utilization two times among women in Ethiopia (AOR = 1.92; 95% CI: 1.53–2.31). Women’s education status was evaluated in seven studies, and six studies reported that the odds of ANC service utilization increased as women’s education increased. The pooled estimates reported that the formal women’s education increased 2.40 times the odds of ANC service utilization (AOR = 2.40; 95% CI: 1.75–3.06) compared to women without formal education (Table 1).

### 3.4. Systematic Review Finding

We could not pool the predictors related to health systems, health care providers, health facilities, and quality of service in the meta-analysis due to insufficient studies. In addition, we found poor consistency and a lack of standardized data categorization in primary studies for a comprehensive meta-analysis. However, we have systematically reviewed a few studies, and the results were presented as follows: the cost, accessibility, and availability of services, the availability of medications, medical supplies, and critical equipment, the attitude or communication of the health care provider, a negative history and experience with health care facilities and systems, and the quality of care are all factors related to health facilities that affect ANC utilization [48,49,50,51,52,53]. Physical coverage of health facilities is still a major problem, especially in Ethiopia’s rural areas. One in three women lives more than five kilometers from the closest medical facility in most rural areas [54]. Access to services is difficult due to inadequate road infrastructure and public transportation, especially during obstetric complications. Additionally, the primary mode of transportation—even for laboring mothers—is walking. Because of these factors, low-income women will receive ANC from less experienced medical professionals who are much more accessible in most remote areas [55,56,57].

The decision to seek care is heavily influenced by the quality of ANC, which is seen as high-quality care. Additionally, it is linked to a person’s assessment of service delivery, which is heavily influenced by their encounters with the health care system and their knowledge of service [54]. According to several studies, pregnant women’s use of the services available to them during their pregnancy was greatly impacted by their opinion of the quality of ANC service [58,59,60]. Additionally, various studies have found that the following factors are important determinants of ANC: the distance from the health organization, the absence of health insurance, the ability to pay for medical services at the facility, the lack of compassionate and respectful care provider there, the low skill level of health care providers, the information regarding maternal health care, and the amount of time it takes to travel to the health institution [29,58,59].

### 3.5. Publication Bias

We checked the publication bias using a graphical diagnostic method (standard and contour-enhanced funnel plot), demonstrating a symmetric shape (see Supplementary File S9). Thus, we further explored using the regression-based Egger test for the small study effect method, and publication bias was not detected due to a *p*-value > 0.05 (*p* = 0.88).

### 3.6. Effect Modification

We entered the interaction terms in the final meta-regression for all plausible and possible combinations of effect modifiers. We included women’s education and women’s knowledge of pregnancy complications, women’s education and women’s perceived importance of ANC, women’s education and women’s media exposure, women’s education and women’s wealth status, wealth status and media exposure, wealth status and women’s autonomy to decide to see if women’s education modifies the effect of women’s knowledge of pregnancy complications, perceived importance of ANC, media exposure, and wealth status, and if wealth status modifies the effect of media exposure and women’s autonomy to decide. None of the interaction terms was statistically significant, implying the absence of a significant effect modification.

## 4. Discussion

### 4.1. Discussion of Main Findings

Wealth status, women’s and their husbands’ education, older/increasing women’s age, media exposure, maternal knowledge about pregnancy complications, planned pregnancy, women’s autonomy to decide on household health care, and positive husband attitude about the ANC service use were found to be predictors of ANC service use.

This meta-analysis found that women and their husbands’ formal education increased the odds of ANC visits. The possible rationale could be that educated families tend to possess good health-seeking behavior, are more autonomous and economically independent, more confident and positive thinkers, have better job opportunities, and have information on ANC’s benefits to the baby and mother [30,61]. Similar results were reported from reviews in developed countries [62], developing countries [63,64], SSA [65], Iran [66], and Ethiopia [67]. The study conducted in Nigeria identified education status as a major predictor of health inequality of ANC utilization [68].

The current meta-analysis also reported that older/increasing women’s age was significantly associated with ANC utilization. Similar results were reported from the reviews conducted in sub-Saharan Africa (SSA) [65], developing countries [63], and Iran [66]. The association of age with ANC utilization might be explained since younger women have less experience in childbearing, as they may be adolescents or newlyweds and thus more likely ignorant of ANC utilization or have limited awareness of pregnancy complications [65]. Also, the confounding effect of parity might be another explanation because low parity was significantly associated with ANC utilization in the reviewed studies [15,23,25,26,29,41].

The household’s highest wealth rank was significantly associated with ANC utilization. Reviews conducted in developing countries [63], SSA [65,69], West African countries [70], South Asia [71], and Iran [66] documented similar significant positive associations. The likely justification might be indirect and direct non-medical and medical costs related to ANC service influencing ANC utilization in SSA [72,73]. Though ANC services are free in Ethiopia, women still pay out of pocket for indirect costs such as transportation, some laboratory tests and drugs, and food during stays in towns. Researchers argued that women from scarce resource communities had been challenged to pay for health care, and these costs present economic barriers to utilizing ANC by pregnant women [74,75]. Therefore, due to the lack of economic access, the mothers may not visit ANC at all, which decreases the number of recommended ANC follow-ups or even starts ANC in late pregnancy.

In this meta-analysis, women’s autonomy in decision-making on household health care was significantly associated with ANC utilization. Similar results were reported from reviews in Bangladesh [76], Ethiopia [77], and SSA [65]. The researchers argued that women denied autonomy could not decide to visit ANC service without authorization from their spouses due to cultural norms and economic dependence [78]. Another possible justification might be that autonomous women, more likely educated, increased their knowledge about the benefits of ANC utilization.

This meta-analysis also showed that women whose pregnancies were planned were associated with ANC use. This result agreed with reviews conducted in developing countries [66], Ethiopia [77], SSA [65], Iran [66], and developing and developed countries [79]. Women who plan to have children may better know their health and baby [65]. Consequently, they may be organized in all the required prearrangements to utilize ANC services efficiently and effectively. Also, women whose pregnancy was unplanned might panic about unintended pregnancy’s social ramifications or implications and thus can avoid ANC services use.

Women’s exposure to mass media was significantly associated with ANC service use. Reviews conducted in developing countries [63] and SSA [65] reported similar significant positive associations. The WHO report argued that women with a high standard of living and income could have better exposure to mass media, increasing awareness and knowledge of ANC services [80]. This argument is supported by results from studies in low-income countries [81,82].

Moreover, this meta-analysis showed that good maternal knowledge about pregnancy complications was significantly associated with ANC service use. Reviews conducted in developing countries [63], SSA [65], and Ethiopia [77] reported similar significant positive associations. The reason might be that women with good knowledge about ODS may better perceive the severity of problems and be prepared to utilize ANC services. Also, many researchers argued that women who have a poor understanding of ODS are less prepared for birth and complications; as a result, they frequently delay seeking proper MHS [83,84].

### 4.2. Limitations of the Study

This review has several strengths. From these, we registered the systematic review in the PROSPERO prospective register with the registration number CRD42022322940 to avoid duplication. Second, our sample size was significantly higher compared to any previous reviews. Hence, the findings are generalizable to all women of reproductive age in Ethiopia and vital to developing appropriate intervention and policy strategies to promote ANC services effectively. Lastly, it comprehensively mapped out the several predictors of ANC service use among WRA in Ethiopia.

On the other hand, the current review has some limitations to consider when presenting our results. First, the probability of recall bias might be high in our review because most included primary studies collected data from the study subject who gave birth in the last five years of preceding surveys. Also, reporting bias may have influenced the findings because data were collected using the respondents’ self-report in most primary studies. There is the risk of intentionally misreporting personally related predictors like age, income, occupation, and household materials (social desirability bias). The extent of these predictors might have been undervalued. The correlation of these predictors with ANC service use might have been underestimated. Second, our review included only two cohort studies; more than 90% is cross-sectional, which restricts us from establishing a causal association.

Nevertheless, the significance of these significant associations cannot be disregarded by the challenge of guaranteeing temporality alone. Measuring the results using various techniques and comparing the results for consistency with other findings are two ways to increase the findings’ credibility [85]. Thus, ANC utilization was linked to predictors that were primarily assessed in the meta-analysis, like wealth status, women and their husband’s education, older/increasing women’s age, media exposure, maternal knowledge about pregnancy complications, and planned pregnancy, demonstrating the consistency of associations with prior research.

Third, we failed to report the pooled effect size for all variables due to the lack of a standardized cut-off point and categorization of data to carry out comprehensive Meta-Analyses. Fourth, the socio-demographic predictors were the most examined and identified predictors of ANC service use in the primary studies. This limited or influenced us from comprehensively exploring predictors such as health service availability and accessibility, health system and health care providers, and behavioral and infrastructure-related factors, which are an area of future research.

### 4.3. Practical Implications of This Study

This research will contribute to the field of public health, particularly in improving ANC utilization, by providing valuable information for implementers, managers, policymakers, and researchers. In addition, this study is essential because it comprehensively and adequately addressed most predictors of ANC utilization to design suitable strategies to promote antenatal care utilization. Furthermore, the potential bodies (target audience) that might be interested in this study are organizations/institutions/health facilities, government officials/managers/policymakers, implementers, researchers, and other stakeholders; partners and bilateral organizations will use findings to make decisions on ANC utilization. Finally, research findings significantly impact the broader community or society by improving ANC utilization in Ethiopia.

## 5. Conclusions

Our review resolved controversies of primary studies and identified several pertinent predictors of ANC service use. Wealth status, women and their husband’s education, older/increasing women’s age, media exposure, maternal knowledge about pregnancy complications, planned pregnancy, women’s autonomy to decide on household health care, and positive husband attitude about the ANC service use were found to be predictors of ANC service use. Based on the results of this review, the intervention strategies in Ethiopia should emphasize the following essential areas: Promoting women’s and husbands’ education status through cooperation with education sector, designing young women’s health education programs to support and mobilize young women about ANC service, creating women-focused economic reforms, developing programs to alter favorable husband’s attitudes to participate in ANC, empowering women to decide on household issues, increasing women exposure to mass media (radio and television), advocating ANC in mass media, and developing programs to improve knowledge about pregnancy complication.

Furthermore, the government and stakeholders should implement women-focused economic reforms, such as boosting women’s participation in rural saving and credit co-operative organizations and productive safety net programs, thereby increasing rural women’s income. Moreover, the government should strengthen existing pregnant women forums that increase women’s knowledge about pregnancy complications and the benefits of ANC to promote ANC utilization in rural settings.

This review and meta-analysis comprehensively addressed several predictors, but health service availability and accessibility, health systems and health care providers, and behavioral and infrastructure-related factors were not adequately investigated. Thus, future researchers should carry out mixed-methods studies to investigate the predictors associated with ANC utilization related to the health system, health facilities, service quality, and health care professionals in rural areas of Ethiopia. Additionally, research on federated learning in smart health care provides insightful information about privacy-preserving methods for health care data analytics that might be used to enhance antenatal care service use and prediction in settings with limited resources [86]. The important but frequently disregarded mental health aspect of maternal care should be investigated, and research on the relationship between physical exercise and mental health prevention offers insight into possible interventions that may be incorporated into all-inclusive ANC programs.

## Figures and Tables

**Table 1:** Meta-analyses of antenatal care utilization predictors among women of reproductive age in Ethiopia, 2023.

Predictors	No of Included in the Analysis	Categories	Pooled AOR with 95% CI
Place of residence	12	urban	1.02 (0.82–1.21)
		rural	1
Wealth rank	6	Highest	1.92 (1.53–2.31)
		Lowest	1
Women’s education status	7	Have formal	2.40 (1.75–3.06)
		No schooling	1
husband’s education status	8	Have formal	1.49 (1.31–1.66)
		No schooling	1
Women age groups	7	≥20 years	1.75 (1.47–2.17)
		<20 years	1
Marital status	5	Married	0.97 (0.69, 1.25)
		Others	1
Parity	6	1–4	0.91 (0.76–1.06)
		>4	1
Media exposure	7	Yes	1.44 (1.21–1.66)
		No	1
Knowledge about pregnancy complications	6	Adequate	1.49 (1.11–1.88)
		Inadequate	1
Planned pregnancy	10	Yes	1.59 (1.28–1.91)
		No	1
women’s autonomy to decide	4	Yes	1.42 (1.23–1.62)
		No	1
Perceived the importance of ANC follow-up	4	Yes	2.68 (0.27–5.09)
		No	1
Husband’s attitude	5	Positive	1.42 (1.23–1.62)
		Negative	1

1: Shows the reference categories.

## Data Availability

The original contributions presented in this study are included in the article/Supplementary Material. Further inquiries can be directed to the corresponding authors.

## Supplementary Materials

The following supporting information can be downloaded at https://www.mdpi.com/article/10.3390/jcm14072517/s1, Supplementary File S1: Search strategy; Supplementary File S2: PRISMA checklist; Supplementary File S3: NOS score; Supplementary File S4: Abstracted data from included studies; Supplementary File S5: Excluded studies from review; Supplementary File S6: Review flow diagram; Supplementary File S7: Features of included studies; Supplementary File S8: Publication bias; Supplementary File S9: Funnel Plot; Supplementary File S10: Datasets.

## Abbreviations

The following abbreviations are used in this manuscript:

ANC	Antenatal Care
AOR	Adjusted Odds Ratio
CI	Confidence Interval
C.S	Cross-sectional
EDHS	Ethiopian Demographic and Health Survey
HCPs	Health Care Providers
H.F	Health Facilities
KA-HDSS	Kilite-Awlaelo Health and Demographic Surveillance System
K.M	Kilometer
LMIC	Low and Middle-Income Countries
MHS	Maternal Health Service
MHSU	Maternal Health Service Utilization
NOS	Newcastle–Ottawa scale
ODS	Obstetric Danger Signs
PNC	Postnatal Care
PRISMA	Preferred Reporting Items for Systematic Review and Meta-Analyses
SBA	Skilled Birth Attendant
SDG	Sustainable Development Goal
SNNPRS	South Nations Nationalities and Peoples Regional State
WHO	World Health Organization
WRA	Women of Reproductive Age
