# Supplementary material for: Predictors of Antenatal Care Service Utilization Among Women of Reproductive Age in Ethiopia: A Systematic Review and Meta-Analysis
Source: J Clin Med. 2025 Apr 7;14(7):2517. doi: 10.3390/jcm14072517 (PMC11989362; doi:10.3390/jcm14072517)
Supplement: Supplementary file 1 [file jcm-14-02517-s001.zip › Supplementary file 1.pdf]

Search: **((((((((((Antenatal care) OR (Maternal health care)) OR (Prenatal care)) AND (Utilization)) OR (Utilisation)) AND (Predictors)) OR (Determinants)) OR (Associated factors)) AND (Women of reproductive age)) AND (Ethiopia)**

((((("prenatal care"[MeSH Terms] OR ("prenatal"[All Fields] AND "care"[All Fields]) OR "prenatal care"[All Fields] OR ("antenatal"[All Fields] AND "care"[All Fields]) OR "antenatal care"[All Fields] OR ("maternal health services"[MeSH Terms] OR ("maternal"[All Fields] AND "health"[All Fields] AND "services"[All Fields]) OR "maternal health services"[All Fields] OR ("maternal"[All Fields] AND "health"[All Fields] AND "care"[All Fields]) OR "maternal health care"[All Fields]) OR ("prenatal care"[MeSH Terms] OR ("prenatal"[All Fields] AND "care"[All Fields]) OR "prenatal care"[All Fields])) AND ("statistics and numerical data"[MeSH Subheading] OR ("statistics"[All Fields] AND "numerical"[All Fields] AND "data"[All Fields]) OR "statistics and numerical data"[All Fields] OR "utilization"[All Fields] OR "utilisation"[All Fields] OR "utilisations"[All Fields] OR "utilise"[All Fields] OR "utilised"[All Fields] OR "utilises"[All Fields] OR "utilising"[All Fields] OR "utilities"[All Fields] OR "utility"[All Fields] OR "utilizations"[All Fields] OR "utilize"[All Fields] OR "utilized"[All Fields] OR "utilizer"[All Fields] OR "utilizers"[All Fields] OR "utilizes"[All Fields] OR "utilizing"[All Fields])) OR ("statistics and numerical data"[MeSH Subheading] OR ("statistics"[All Fields] AND "numerical"[All Fields] AND "data"[All Fields]) OR "statistics and numerical data"[All Fields] OR "utilization"[All Fields] OR "utilisation"[All Fields] OR "utilisations"[All Fields] OR "utilise"[All Fields] OR "utilised"[All Fields] OR "utilises"[All Fields] OR "utilising"[All Fields] OR "utilities"[All Fields] OR "utility"[All Fields] OR "utilizations"[All Fields] OR "utilize"[All Fields] OR "utilized"[All Fields] OR "utilizer"[All Fields] OR "utilizers"[All Fields] OR "utilizes"[All Fields] OR "utilizing"[All Fields])) AND ("predictor"[All Fields] OR "predictors"[All Fields])) OR ("analysis"[MeSH Subheading] OR "analysis"[All Fields] OR "determination"[All Fields] OR "determinant"[All Fields] OR "determinants"[All Fields] OR "determinate"[All Fields] OR "determinated"[All Fields] OR "determinates"[All Fields] OR "determinating"[All Fields] OR "determinations"[All Fields] OR "determine"[All Fields] OR "determined"[All Fields] OR "determines"[All Fields] OR "determining"[All Fields]) OR (("associate"[All Fields] OR "associated"[All Fields] OR "associates"[All Fields] OR "associating"[All Fields] OR "association"[MeSH Terms] OR "association"[All Fields] OR "associations"[All Fields]) AND ("factor"[All Fields] OR "factor s"[All Fields] OR "factors"[All Fields]))) AND (("womans"[All Fields] OR "women"[MeSH Terms] OR "women"[All Fields] OR "woman"[All Fields] OR "women s"[All Fields] OR "womens"[All Fields]) AND ("reproduction"[MeSH Terms] OR "reproduction"[All Fields] OR "reproductions"[All Fields] OR "reproductive"[All Fields] OR "reproductively"[All Fields] OR "reproductives"[All Fields] OR "reproductivity"[All Fields]) AND ("age"[Journal] OR "age omaha"[Journal] OR "age dordr"[Journal] OR "adv genet eng"[Journal] OR "age"[All Fields])) AND ("ethiopia"[MeSH Terms] OR "ethiopia"[All Fields] OR "ethiopia s"[All Fields])

Table 1: The systematic PubMed search method and limited by utilizing English language and studies done between 2002 to 2022 years.

Search #	Query	Results	Time
11	(((((((((#1) OR (#2)) OR (#3)) AND (#4)) OR (#5)) AND (#6)) OR (#7)) OR (#8)) AND (#9)) AND (#10)	**1,011**	15:01:47
10	Ethiopia	30,825	14:59:39
9	Women of reproductive age	112,473	14:59:09
8	Associated factors	1,860,911	14:58:43
7	Determinants	########	14:58:26
6	Predictors	450,350	14:58:04
5	Utilisation	4,048,246	14:57:37
4	Utilization	4,048,246	14:57:21
3	Prenatal care	54,227	14:56:53
2	Maternal health care	105,643	14:56:20
1	Antenatal care	65,564	14:55:23

Table 2: The systematic Medline search method and limited by utilizing English language and studies done between 2002 to 2022 years.

Search #	Searches	Results	Search type	Actions
1	Antenatal care	6288	Advanced	Display
2	Maternal health care	12723		
3	Prenatal care	21534		
4	1 or 2 or 3	39652		
5	Utilization	15672		
6	Determinants	14356		
7	Associated factors	33564		
8	4 and 5	1547		

9	4 or 6	162895		
10	4 and 7	298		
11	Ethiopia	9569		
12	8 or 9	164987		
13	9 and 10	281		
14	12 and 13	243		
15	4 or 5 or 6 or 7	33678		
16	11 and 15	812		
17	8 or 9 or 10	162145		
18	11 and 17	612		
19	4 and 11	330		
20	18 or 19	619		
21	18 and 19	512		
22	20 and 21	**512**		

Table 3: The systematic CINAHL search method and limited by utilizing English language, age and studies done between 2002 to 2022 years.

Search ID#	Search Terms	Search Options	Last Run Via	Results
S7	S1 AND S3 AND S4 AND S65	Search modes - Boolean/Phrase	Interface - EBSCOhost Research Databases Search Screen - Advanced Search Database - CINAHL Complete	**211**
S6	S1 AND S3 AND S4 AND S5	Search modes -	Interface -	211

		Boolean/Phrase	EBSCOhost Research Databases Search Screen - Advanced Search Database - CINAHL Complete	
S5	Ethiopia	Search modes - Boolean/Phrase	Interface - EBSCOhost Research Databases Search Screen - Advanced Search Database - CINAHL Complete	3,557
S4	Associated factors OR Factors associated OR Influencing factors OR Predictors OR Predicting factors OR (Correlates or determinants) OR (Determinants or factors) OR (risk factors or protective factors)	Search modes - Boolean/Phrase	Interface - EBSCOhost Research Databases Search Screen - Advanced Search Database - CINAHL Complete	1,155,068
S3	S1 OR S2	Search modes - Boolean/Phrase	Interface - EBSCOhost	29,145

			Research Databases Search Screen - Advanced Search Database - CINAHL Complete	
S2	Utilization OR utilisation	Search modes - Boolean/Phrase	Interface - EBSCOhost Research Databases Search Screen - Advanced Search Database - CINAHL Complete	13,568
S1	(antenatal care or prenatal care) OR First antenatal care OR Antepartum care OR ANC OR PNC OR Antenatal follow up OR Antenatal period OR Antenatal service OR Antenatal care visit OR Antenatal presentation OR Antenatal attendance	Search modes - Boolean/Phrase	Interface - EBSCOhost Research Databases Search Screen - Advanced Search Database - CINAHL Complete	298,676

Table 2: The systematic EMBASE search method and limited by utilizing English language and studies done between 2002 to 2022 years.

Search #	Searches	Results
1	Antenatal care	132377
2	Maternal health care	12723
3	Prenatal care	21534
4	First antenatal care	705334
5	Antepartum care	342066
6	Antenatal follow up	485524
7	Antenatal period	304064
8	Antenatal service	311668
9	Antenatal care visit	131988
10	Antenatal presentation	28661
11	Antenatal attendance	795139
12	1 or 2 or 3 or 4 or 5 or 6 or 7 or 8 or 9 or 10 or 11	2244549
13	Utilization	134573
14	utilisation	133994
15	13 or 14	2867897
16	Determinants	154332
17	Associated factors	117675
18	Influencing factors	7930
19	Predicting factors	261656
20	Correlates	182554
21	Risk factors	962833
22	16 or 17 or 18 or 19 or 20 or 21	2073703
23	Ethiopia	13770
24	12 and 15 and 22 and 23	**1123**
