# Supplementary material for: Predictors of Antenatal Care Service Utilization Among Women of Reproductive Age in Ethiopia: A Systematic Review and Meta-Analysis
Source: J Clin Med. 2025 Apr 7;14(7):2517. doi: 10.3390/jcm14072517 (PMC11989362; doi:10.3390/jcm14072517)
Supplement: Supplementary file 1 [file jcm-14-02517-s001.zip › Supplementary file 4.pdf]

Additional file 3: The quality of studies included in systematic review and Meta-Analysis based on NOS checklist evaluation

Author and year	Subject selection (4 stars)	Groups comparability (2 stars)	Outcome measurement (3 stars)	Quality	Predictors utilized for Meta-analysis
Kidist B et al, 2013	4	2	3	High	**Women age:** > = 20- R and 15-19 =2.04(0.33–12.74), **women education**: No formal-R and have formal = 2.65 (1.08–6.49), **media exposure**: No-R and yes = 4.42 (1.09 – 17.78), **planned pregnancy:** No-R and yes = 2.97 (1.18-7.46), **knowledge on ODS**: poor-R and good = 3.54 (1.24-10.18)
Tesfaye G, et al, 2018	3	2	3	High	**Women education status:** No formal-R and have formal = 3.02 (1.26, 7.25), **previous utilization of ANC:** No-R and yes = 20 (14.28-33.33), **wealth index**: lowest-R and highest = 6.87 (1.81, 26.01), **husband attitude about ANC:** negative/neutral-R and positive = 3.70 (1.61 -9.09), **knowledge of ODS:** No-R and yes = 1.92 (1.27- 2.86), **perceived importance of ANC visit:** Not important-R and Important = 1.89(1.17, 3.06)
Jira C, 2005	2	1	2	Low	**Planned pregnancy:** yes = 1.18 (0.31–4.52) and No-R, **perceived importance of ANC visit:** not-R and yes = 7.08 (1.13, 44.29)
Desalew ZA et al, 2014	3	1	3	High	**Women education**: no formal-R and have formal- 2.59 (1.09–6.15), **residence**: rural-R and urban = 5.46(1.99–14.95), **perceived importance of ANC visit:** not-R and Yes = 10.17 (2.77, 37.00)
Bontu F, 2007	2	1	2	Low	**Women age:** > = 20-R and 15-19- 2.74 (1.38–5.43), **marital**

					**status:** married= 0.74(0.42–1.31 and others-R, **parity**->4-R and 1-4= 1.75(0.50-6.15)
Abebaw GW et al, 2013	3	2	3	High	**Women education**: No formal-R and have formal = 1.26 (0.98–1.62), **husband education**: no formal- R and have formal- 1.22 (0.77–1.94), **planned pregnancy**: No-R and yes = 1.27 (0.82–1.96), **knowledge on ODS**: poor-R and good- 1.35 (0.96, 1.89), **wealth quintile:** poorest-R and richest = 0.83 (0.54, 1.27), **previous pregnancy ANC use-** No-R and yes = 3.39 (1.98, 5.80)
Gebeyeu TN et al, 2015	3	1	2	Low	**Women education**: no formal-R and formal = 3.171 (1.480-6.792), **women occupation status**: housewife-R and others- 6.345 (2.718-14.811), **wealth status:** poorest-R and richest = 3.35 (1.334-8.413), **perceived important of ANC:** no-R and yes = 4.314 (1.371- 13.577), **plan of last pregnancy:** No-R and yes- 6.333 (2.454- 16.340), **decision on ANC utilization**: husband only-R and Both = 3.507 (1.837-6.694)
Yohannes AM et al, 2014	3	2	3	High	**Women education status**: No formal-R and formal = 1.62(1.25-2.10), r**esidence:** rural-R and urban = 2.20(1.25-3.87), **women age**: > = 20-R and 15-19 = 0.50 (0.40-0.63), **marital status**: Others- R and married = 1.45(0.87-2.42)
Yalem T et al, 2013	3	2	3	High	**Women education**: No formal-R and have formal = 1.45 (1.05-2.00), **marital status-** others-R and married = 2.57(1.44–4.58), **parity**:1-4-R, 5-7=1.16 (0.88-1.55) and 8-11= 1.28 (0.87-1.88)

Zeine A et al, 2010	2	2	2	Low	**Women education**: No formal-R and have formal- 0.68(0.13–3.58), **residence**: rural-R and urban-0.39 (0.13–1.18), **husband's attitude about ANC:** No-R and yes = 1.24 (0.46, 3.32), **planned pregnancy:** No-R and yes = 1.76 (1.1, 2.8), **knowledge of ODS:** poor-R and good-12.9 (7.6, 21.9)
Abebaw AM et al, 2021	4	2	3	High	**Husband education:** No formal-R and have formal = 7.69 (2.86-20), **wealth index**: poorest-R and richest = 2.10 (1.26, 3.50)
Zeleke D et al, 2015	3	2	2	High	**Women education**: No formal-R and have formal =1.68 (0.96–2.94), **husband education:** No formal- R and have formal = 1.52(0.88–2.62)<br>**Women age:** > = 20-R and 15-19 = 1.1 (0.30, 4.06), **parity**: parity >4 –R and parity 1–4 2.62(1.56-4.40), **planned pregnancy**: No–R and yes = 1.96 (1.10–3.60), **knowledge of ODS:** Poor-R and good = 7.0 (3.8,13.0)
Melese G et al,2016	3	2	2	High	**Residence**: rural-R and urban = 1.01(0.04–28.72), **women education**: No formal-R and have formal = 1.32(0.49-3.58), **husband education**: No formal-R and have formal = 1.61 (0.60-4.35), **previous pregnancy ANC use:** No-R and Yes = 0.87 (0.30-2.49), **media exposure:** No-R and Yes = 5.66 (1.46, 21.94)
	3	2	2	High	**Women education:** No formal-R and have formal = 3.90 (2.27-6.71), **residence**: rural-R and urban = 2.11(1.01-4.42), **pregnancy planned:** No-R and Yes = 4.14 (2.18 - 7.86),

					**knowledge on ODS:** No-R and Yes = 1.58(0.95-2.63)
Shegaw M et al, 2014	3	2	2	High	**Residence**: rural-R and urban 2.3(1.90 -2.90), **marital status**: Others-R and married = 0.9 (0.55-1.46), **women education status**: No formal-R and have formal = 2.39 (1.72-3.33), **husband education**: No formal-R and have formal = 1.60(1.36–1.88), **women age:** > = 20-R and 15-19 = 0.80 (0.60 -1.28), **parity:** > = 5 -R and 1–4 = 0.83(0.66-1.04), **wealth index**: Poorest-R and richest = 3.70 (2.90-4.80), **autonomy of woman:** No-R and Yes = 1.40 (1.2-1.6), **media exposure:** No-R and Yes = 1.30 (1.30-2.00)
Muluwas A et al, 2015	3	2	2	High	**Women education status**: No formal-R and Yes = 3.24 (1.84 - 5.72), **residence:** rural-R and urban = 3.70 (0.83 -16.43)
Gurmesa T G,2009	3	1	2	Low	**Residence**: rural- R and urban = 1.51 (1.22-2.78), **women education**: No-R and have formal = 6.25(1.49-27.39), **husband education status**: No-R and have formal = 1.56 (1.11-2.89)
Kassahun T et al, 2019	4	2	3	High	**Education level:** No formal-R and yes- formal = 2.16 (1.42-3.29), **residence**: rural-R and urban – 2.36 (1.21 – 4.64), **wealth status**- poorest-R and richest- 2.20 (1.45-3.37)
Tsegay B et al, 2021	4	2	3	High	**Wealth index:** poorest-R and richest -0.17 (0.07, 0.42), **women education**- No-R and Yes = 4.72 (2.82, 7.90), **have plan on current pregnancy**- No-R and Yes- 3.65 (1.67, 8.01)
N Regassa,	3	1	2	Low	**Women education**: No formal-R and have formal-1.39 (1.02-

2011					1.93), **planned pregnancy:** No-R and Yes = 2.17 (1.56-3.02), **media exposure:** No-R and Yes = 3.72 (1.32-5.61)
Nejimu B et al, 2016	2	2	2	Low	**Place of residence:** urban**-** 2.224 (1.376, 3.595) and rural-R, **wealth index**: poorest-R and richest = 1.679 (1.104, 2.552), **media exposure:** No-R and yes = 3.134 (2.204, 4.457)
Wubareg S et al, 2017	3	1	2	Low	**Maternal age:** less than equal to 20-R and greater 20-0.28(0.21-0.46), **attitude:** Unfavorable attitude-R and favorable attitude = 4.8(3.5-9.0), **parity**: One- 5.9(3.8-7.2) and greater than 4-R, **husband education:** No-R and have formal = 0.67 (0.49-0.73)
Bahilu T et al, 2009					**Presence of husband approval:** Yes-8.01(4.57-14.06) **and** No-R**, Educational Status of women:** No formal-R and have formal = 3.90 (2.27 - 6.71)**; Age at first pregnancy:** <=20- 2.94(1.66,5.20), >20-R**; Residence:** Urban-2.11(1.01-4.42), Rural-R; **Did you plan your last pregnancy:** Planned = 4.14(2.18-7.86) and Unplanned-R**, Know danger signs of pregnancy:** Yes = 1.58 (0.95-2.63) and No-R
