# Supplementary material for: Predictors of Antenatal Care Service Utilization Among Women of Reproductive Age in Ethiopia: A Systematic Review and Meta-Analysis
Source: J Clin Med. 2025 Apr 7;14(7):2517. doi: 10.3390/jcm14072517 (PMC11989362; doi:10.3390/jcm14072517)
Supplement: Supplementary file 1 [file jcm-14-02517-s001.zip › Supplementary file 5.pdf]

Abstracted AOR and 95% CI

S.No	First Author and Year	Variables
1	Tesfaye G, et al, 2018	**Women education status:** No formal-R and have formal = 3.02 (1.26, 7.25), **previous utilization of ANC:** No-R and yes = 20 (14.28-33.33), **wealth index**: lowest-R and highest = 6.87 (1.81, 26.01), **husband attitude about ANC:** negative/neutral-R and positive = 3.70 (1.61 -9.09), **knowledge of ODS:** No-R and yes = 1.92 (1.27- 2.86), **perceived importance of ANC visit:** Not important-R and Important = 1.89(1.17, 3.06)
2	Kidist B et al, 2013	**Literacy status -** No schooling- R , Schooling- 2.65(1.08-6.49)**; Occupation of the respondent -** Housewife- 1.81(0.78-4.22) **,** Others (civil servant students, maid servant, merchant etc…) **; Average family monthly income -** <23$- 0.15(0.25-0.87)- 1.46(0.25-0.87) **,** 23-57$-0.25(0.04-1.47)- 0.25(0.04-1.47)**,** Above 57$ - R**; Marital status** - Married - 1.07 (0.40- 2.84), Others(divorced, widowed, never married)- R; **Planned pregnancy -** Yes- 2.94(1.15-7.53), No– R**; Knowledge on danger signs of pregnancy -** Better Knowledge – 3.54(1.24-10.15)**,** Poor Knowledge; **Age at last birth** - 15-19-2.04(0.33-12.74), >=20- R; **Parity** 1-4-0.81(0.32- 2.06), >5 – R; **Media exposure -** No exposure –R**,** Radio or TV-4.01(1.49-10.83)**,** Radio and TV- 4.42(1.10-17.79); **Presence of husband approval on ANC -** Yes-9.00(3.71-21.86)**,** No- 9.00 (3.71- 21.86)
3	Zeine A et al, 2010	**Residence-** Urban-0.39(0.13-1.17)**,** Rural-R**; Educational status-** No education - R**,** Primary school and above- 0.68(0.13-3.58)**;**Age**-** <20- 0.83(0.27-2.53), 20-24-0.27(0.09-0.73)**,**25-29-0.32(0.16-0.62), 30-34-0.62(0.34-1.15)**;Ever had abortion**- Yes-1.13 (0.57, 2.24), No-R**; Family size**- 1-2-8.14(1.82-36.44), 3-4-1.13(0.53-2.43),>=5-R**; Positive husband attitude** - Yes-1, No-1.24(0.46-3.32)**; Religion-** Protestant-R**,** Muslim-0.55(0.22-1.40)**,** Orthodox-0.68(0.29-1.60)**,** Others-4.93(1.6-15.19)
4	Muluwas A et al, 2015	**Place of residence-** Urban -3.70(0.83-16.43), Rural-R **; Educational status-** Illiterate – R , Literate 3.24(1.84-5.72)**; Availability of TTBAs in Kebele-** No- R**,** Yes- 2.21(1.19-4.12)**; Knowledge on ANC service -** Knowledgeable -1.96(1.04-3.68), Not-Knowledgeable 1**; Knowledge on delivery service-** Not-Knowledgeable**-**R, Knowledgeable-1.55(0.8-3.0), **Religion-** Orthodox -R**,** Muslim-1.46(0.69-3.07) **,** Others- 0.53(0.07-4.22)**; Ethnicity-**Berta- R, Amhara -0.28(0.13-0.62)**,** Oromo-1.13(0.13-9.63)**,** Others-0.08(0.02-0.39) **; Attitude towards ANC -** Unfavourable attitude- R , Favourable attitude-1.95(0.79-4.78)**; Availability delivery service-** Not available-R, Available - 0.93(0.45-1.91)**; Transportation –** No ,Yes-1.8(0.58-5.62)**; Time taken by the foot from home to health facility** - <30min-R ,>=30min-0.25(0.11-0.58)
5	Abebaw AM et al, 2021	**Husband education:** No formal-R and have formal = 7.69 (2.86-20), **wealth index**: poorest-R and richest = 2.10 (1.26, 3.50)
6	Yohannes AM et al, 2014	**Marital status-** Married-1.45(0.87-2.42) , Others(Single, dissolved)- R **; Age (Years)** – 15-19-0.50(0.40-0.63), >=20-R**; Maternal educational status -** No education -R**,** Educated -1.62(1.25-2.10)**; Maternal occupation-** Farmer-2.31(1.23-4.35)**,** Daily labourer -1.36(0.74-2.51)**,** Housewife 1.09(0.66-1.78)**,** Student -1.05(0.55-2.02)**,** Others-R**; Residence-** Urban -2.20(1.25-3.87)**,** Rural-R
7	Zeleke D et al, 2015	**Mother's education -** No education- R**,** Primary and above -1.68(0.96-2.94); **Employment status-** Unemployed –R**,** Employed for cash-1.7(0.8-3.3), Employed for non-cash-2.7(0.6-12.5);**Husband education -** No education- R**,** Primary and above -1.52(0.88-2.62); **Age group of women-** 15-19- **1.1** (0.30, 4.06)**, >=20-**R; **Women's autonomy-**Higher- 1.6 (0.8, 2.9), Lower- R; **Average family monthly income-** <450- 0.7(0.3-1.7)**,** 450-1100-1.1(0.5-2.3), >1100 –R; **Parity-** Parity 1-4 2.62(1.56-4.40), Parity >4 –R; **Pregnancy intention-** Intended -1.90(1.01-3.59), Unintended –R; **Aware danger signs of pregnancy-** Yes-7.0(3.8-13.0), No – R
8	Jira C, 2005	**Occupation of women-** Housewife- R, Housemaid-1.97(0.30-12.91), Student-5.34(1.12-25.64)**,** Government Emp-0.00(0.00-2.07)**,** Merchant-1.89(0.34-10.64) **,** Daily labourer-0.00(0.00-2.69)**; Husband Attitude towards ANC-** Positive-R , Negative-4.77(0.96-23.75); **Religion-** Muslim-R, Orthodox-2.68(0.96-7.46), Catholic- 2.38(0.39-14.60),Protestant-0.50(0.04-6.05) **; Women's understanding the importance of ANC -**Very important -1.65(0.54-5.03), Important – 18.10(1.85-177.49)**; Pregnancy intention** –Yes-1.18(0.31-4.52) , No-R

9	Kassahun T et al, 2019	**Education level:** No formal-R and yes- formal = 2.16 (1.42-3.29), **residence**: rural-R and urban – 2.36 (1.21 – 4.64), **wealth status**- poorest-R and richest- 2.20 (1.45-3.37)
10	Melese G et al,2016	**Residence-** Urban- 1.01(0.04-27.06), Rural-R**; ANC follow up for previous pregnancy-** Yes - 0.87 (0.30, 2.49), No- R**; Awareness on places to get skilled providers for ANC-** Yes - 51.55 (13.92, 190.97), No-R**; Skilled personnel preferred for ANC services-** Yes- 11.00 (3.02, 40.04), No- R**; Maternal education -** Education-1.32(0.49-3.58), No education-R**; Listening to radio-** Yes -5.66(1.46-21.94), No**; Complications during previous pregnancies or births-** Yes- 1.12 (0.32, 3.89), No-R**; Husband education -**Education- 1.61 (0.60, 4.35), No education- R**; Main road to nearest HF-** Yes- 0.81 (0.22, 3.01), No-R**; Distance of WHDT from nearest HF with skilled care (KMs)-** <=2km-8.18(1.08-62.20), 3-5km-0.51(0.15-1.67), >5km – R
11	N Regassa, 2011	**Children ever born** - 1-3 children-R, 4-6 children- 0.83(0.60-1.15), 7-10 children- 0.79(0.57-1.10); **Religion**- Orthodox Christian –R, Catholic-0.91(0.42-2.14), Protestant-0.41(0.31-1.93), Muslim-0.98(0.51-2.10) , Traditional-1.12(0.69-1.73) , Others-0.73(0.38-1.89); **Radio listening frequency**- Almost every day-R, Twice a week-0.49(0.36-.68), Once in a fortnight-0.27(0.20-0.37), Not at all-0.94(0.19-1.29); **Age of the women**- 15-24 -(R) Age 25-34- 0.57(0.41-0.79) Age 35-49-0.37; **Pregnancy reaction**- Wanted-2.17(1.56- 3.02) , Unwanted-R ; **Usual work**- Self-employment-R, Civil servant-1.96(1.24-3.6), Farmer- 0.54(0.32-1.95), Petty trader- 1.26, Others-0.27; **Women's literacy status**- Literate-1.39(1.01-1.92), Illiterate –R
12	Shegaw M et al, 2014	**Husband's work status-** Jobless – , Working -1.1(1.1-1.3)**; Woman's work status-** Jobless, Working- 1.1(1.01-1.3)**; Marital status-** Others(Never married, Divorced/separated/widowed)- R, Married-0.9(0.55-1.46) **; Religion -** Orthodox -1.3(0.8-2.1), Catholics -2.2(0.8-3.5), Protestant-1.7(0.9-2.7) , Muslims-1.5(0.8-2.2), Others-R; **Age-** 15-19- 0.80(0.60, 1.28), **>=**20-R**; Educational status-** No education – R, Primary and above - 2.39(1.72-3.33) **Ethnicity-** Amhara - 1.9(1.4-2.1), Gurage -3.1(2.2-5.4), Oromo-1.2(1.1-1.4), Sidama- 0.6(0.5-1.0), Tigrae- 2.7(1.9-3.1) , Wolyita- 0.4(0.3-0.7), Others –R; **Residence-** Urban 2.3(1.81-2.92), Rural-R**; Number of births in the last 5 years**- 1 birth- 1.3(1.1-1.5), more than two birth –R**; Husband education-** No education –R, Primary and above -1.60(1.36-1.88)**; Reading newspaper frequency-** Not at all, Less than once a week-1.1(0.8-1.5), At least once a week-0.9(0.5-1.6)**; Listening radio frequency-** Not at all-R, <1week-1.4(1.2-1.6), At least once a week -1.3(1.1-1.6)**; Watching television frequency-** Not at all- R, <1week-1.3(1.1-1.5), At least once a week- 1.3(1.3-2.0)**; HH Wealth-** Poorest 1, Poorer-1.2(1.1-1.5) , Middle-1.5(1.2-1.8), Richer-1.7(1.4-2.1) , Richest-3.7(2.9-4.8) **; Autonomy of woman -** Women and Husband - 1.4(1.2-1.6), Husband only or others-R**; Parity –** 1-4 -0.83(0.66-1.04), >=5 –R
13	Tsegay B et al, 2021	**Wealth index:** poorest-R and richest -0.17 (0.07, 0.42), **women education**- No-R and Yes = 4.72 (2.82, 7.90), **have plan on current pregnancy**- No-R and Yes- 3.65 (1.67, 8.01)
14	Bahilu T et al, 2009	**Distance travelled in minutes-** <=60Min- 8.80(4.85-15.96), >60-R**; Presence of Husband Approval-** Yes-8.01(4.57-14.06), No-R**; Exposure to; Illness in past pregnancies-** Yes-2.0(1.18-3.71), No**; Perceived Susceptibility in future pregnancies-** Yes - 4.82(2.74-8.45), No**; Educational Status of women-** Illiterate –R, Primary and above-3.90(2.27-6.71)**; Age at first pregnancy-** <=20- 2.94(1.66,5.20), >20-R**; Residence-** Urban-2.11(1.01-4.42), Rural-R**; Did you plan your last pregnancy-** Planned-4.14(2.18-7.86), Unplanned-R**; Know danger signs of pregnancy-** Yes-1.58(0.95-2.63), No-R
15	Yalem T et al, 2013	**Health Facility in village -** No-R, Yes-1.83(1.41-2.38)**; Marital status-** Others(Single or widowed, Divorced)–R, Married-2.57(1.44-4.58) **, ; Education-** No Education-R **,** Primary school and above- 1.45(1.05-2.00); **Husbands Occupation-** Farmer-R, Others-2.26(1.43-3.58)**; Parity-** 1-4-R, 5-7-1.16(0.88-1.55), 8-11-1.28(0.87-1.88)
16	Nejimu B et al, 2016	**Place of residence:** urban**-** 2.224 (1.376, 3.595) and rural-R, **wealth index**: poorest-R and richest = 1.679 (1.104, 2.552), **media exposure:** No-R and yes = 3.134 (2.204, 4.457)
17	Gurmesa TG,2009	**Occupation-** Housewife-R , Others- 0.87(0.32-2.42)**; Place of residence-** Urban- 1.60(0.99-2.58), Rural- R**; Educational status-** No education- R, Educated – 6.25(1.49-26.27)**, ; Husband's occupation-** Farmer- R, Others-1.21(0.66-2.23)**; Have radio-** Yes-2.08(1.37-3.13), No- R**; Husband's education,** No education- R, Educated- 1.66(0.98-2.82)**; Knowledge on ANC-** Knowledgeable –R, Not-Knowledgeable -0.03(0.02-0.05)**; The floor is made up of-** Mud, Cement - 1.02(0.34, 3.12)**; Monthly income (Eth.Birr)-** <500- R, >=500-1.53(1.22-3.52), **The roof is made of**- Thatched-Corrugated sheet- 0.70(0.38, 1.26)

18	Abebaw GW et al, 2013	**Husband education -** No education R, Primary above- 1.28(1.03-1.60)**; ANC in previous pregnancy-** No –R, Yes-3.39(1.98-5.80)**; Awareness on places to get skilled provider-** No-R, Yes-1.63(1.07-2.49)**; Average distance to Nearest HC**- 0.83(0.46-1.48); **Mother's education-** No education R, Primary and above- 1.26(0.98-1.62)**; Wealth quintile-** Lowest – R, Second-1.02(0.69-1.51), Middle-1.14(0.75-1.72), Fourth- 1.03(0.68-1.56), Highest- 0.83(0.54-1.27)**; Main source of income- -** Farming-R, Mixed-1.35(0.47-3.88)**; Health professionals preferred for the care-** Yes - 1.64 (1.14, 2.36), No-R**; Birth Order -** 1 –R, 2-3- 0.75(0.53-1.07), 4-5-0.66(0.44-0.99), 6+- 0.79(0.50-1.25)**; Pregnancy wontedness-** Wanted-1.27(0.82- 1.96), Unwanted-R**; Awareness on risk of pregnancy-** No-R, Yes-1.35(0.96-1.89)
19	Wubareg S et al, 2017	**Maternal age:** less than equal to 20-R and greater 20- 0.28(0.21-0.46), **attitude:** Unfavorable attitude-R and favorable attitude = 4.8(3.5-9.0), **parity**: One- 5.9(3.8-7.2) and greater than 4-R, **husband education:** No-R and have formal = 0.67 (0.49-0.73)
20	Desalew ZA et al, 2014	**Age of respondent-** 15-24-1.45(0.26-8.03), 25-39-5.44(1.13-26.11), 40-49 –R **; Family size-** Below five Children-R, 5+- 1.51(0.73-3.13)**;Perception of quality of services-** Good/very good-10.13(2.78-37.01), Bad or very bad-R**; Educ. Respondent-** Illiterate (RC)-R, Primary and above- 2.59(1.09-6.15)**; History of abortion/still birth-** Yes- 13.54(5.67-32.80), No-R**; Residence -** Urban -5.46(1.13-26.29), Rural-R**; Health educ. on maternal health-** Yes - 3.184(0.472 - 21.47), No-R**; Means of transport to health facility-** Walk-R, Vehicle-2.72(0.92-8.01)
21	Bontu F, 2007	**Occupation -** Housewife-R, Trader-0.26(0.10-0.62), Civil servant- 1.02(0.52-1.98), Government employee – 0.04(0.000-0.23), Other(student farmer)- 4.06(1.50-11.40)**; Marital status -** Others(Single or widowed, Divorced)–R , Married-0.74(0.42-1.31)**; Religion ,** Orthodox-R, Muslim-3.64(1.64-7.08), Catholic – 2.54(0.90-6.86), Protestant- 2.63(1.16-6.06); **Age in years -** 15-19- 2.74(1.38-5.43), >=20-R; **Ethnicity**- Oromo-R, Amhara-1.15(0.58-2.23), Gurage-1.57(0.71-3.33), Other-0.94(0.29-2.55); **Monthly income-** <300-4.24(2.44-7.36), >=300-R; **Parity** :1-4-1.75(0.50-6.15), >4-R
22	Gebeyeu TN et al, 2015	**Women education**: no formal-R and formal = 3.171 (1.480- 6.792), **women occupation status**: housewife-R and others- 6.345 (2.718-14.811), **wealth status:** poorest-R and richest = 3.35 (1.334-8.413), **perceived important of ANC:** no-R and yes = 4.314 (1.371- 13.577), **plan of last pregnancy:** No-R and yes- 6.333 (2.454- 16.340), **decision on ANC utilization**: husband only-R and Both = 3.507 (1.837-6.694)
