# Supplementary material for: Predictors of Antenatal Care Service Utilization Among Women of Reproductive Age in Ethiopia: A Systematic Review and Meta-Analysis
Source: J Clin Med. 2025 Apr 7;14(7):2517. doi: 10.3390/jcm14072517 (PMC11989362; doi:10.3390/jcm14072517)
Supplement: Supplementary file 1 [file jcm-14-02517-s001.zip › Supplementary file 7.pdf]

Excluded studies and the reasons for exclusion

We excluded 40 studies after critically evaluated the full texts based on pre-defined eligibility criteria.

S.No	Study name	Reasons of exclusion
1	Biratu BT, Lindstrom DP. The influence of husbands' approval on women's use of prenatal care: Results from Yirgalem and Jimma towns, south west Ethiopia. Ethiopian Journal of Health Development. 2006; 20(2):84-92.	The data collected before 2002 (Not include the FANC)
2	Ayalew TW, Nigatu AM. Focused antenatal care utilization and associated factors in Debre Tabor Town, northwest Ethiopia, 2017. BMC Res Notes. 2018 Nov 16;11(1):819. doi: 10.1186/s13104-018-3928-y. PMID: 30445991; PMCID: PMC6240228.	The outcome was Focused ANC utilization (women who attended a minimum of four scheduled ANC visits and receiving all the WHO recommended)
3	Ejigu T, Woldie M, Kifle Y. Quality of antenatal care services at public health facilities of Bahir-Dar special zone, Northwest Ethiopia. BMC Health Serv Res. 2013;13:443.	Focus on quality of ANC
4	Gedefaw M, Muche B, Aychiluhem M. Current Status of Antenatal Care Utilization in the Context of Data Conflict: The Case of Dembecha District, Northwest Ethiopia. Open Journal of Epidemiology. 2014;04(04):208-16.	Problem in the findings
5	Kare AP, Gujo AB, Yote NY. Quality of antenatal care and associated factors among pregnant women attending government hospitals in Sidama Region, Southern Ethiopia. SAGE Open Med. 2021 Nov 23;9:20503121211058055. doi: 10.1177/20503121211058055. PMID: 34868590; PMCID: PMC8640313.	Focus on quality of ANC
6	Gela BD. Antenatal Care Utilization and Associated Factors from Rural Health Extension Workers in Abuna Gindeberet District, West Shewa, Oromiya Region, Ethiopia. American Journal of Health Research. 2014;2(4):113.	Include ANC user only from Health extension worker
7	Gudayu TW, Woldeyohannes SM, Abdo AA. Timing and factors associated with first	The study participants were antenatal care user

	antenatal care booking among pregnant mothers in Gondar Town; North West Ethiopia. BMC Pregnancy Childbirth. 2014;14:287.	
8	Belay A, Astatkie T, Abebaw S, Gebreamanule B, Enbeyle W. Prevalence and factors affecting the utilization of antenatal care in rural areas of Southwestern Ethiopia. BMC Pregnancy Childbirth. 2022 Jan 14;22(1):30. doi: 10.1186/s12884-021-04362-8. PMID: 35031008; PMCID: PMC8759251.	The dependent variable was at least 4 ANC visits (≥4 visits) or less than 4 ANC visits
9	Karim AM, Admassu K, Schellenberg J, Alemu H, Getachew N, Ameha A, et al. Effect of Ethiopia's health extension program on maternal and newborn health care practices in 101 rural districts: a dose-response study. PLoS ONE [Electronic Resource]. 2013;8(6):e65160.	Focus on health care practice
10	Getachew T, AbajobirAA,Aychiluhim M. Focused Antenatal Care Service Utilization and Associated Factors in Dejen and Aneded Districts, Northwest Ethiopia. Primary Health Care 2014 4:170.	The aim was on the number of visits rather than ANC utilization
11	Berhane Fseha, Gebrehiwot Gebremariam. Focused Antinatal Care Service Utilization and Associated Factors Among Pregnant Women's in Shire, Tigray, Ethiopia. Biomed J Sci & Tech Res 15(2)-2019. BJSTR. MS.ID.002686.	Due to outcome measurement
12	Materia E, Mehari W, Mele A, Rosmini F, Stazi M, Damen H, et al. A community survey on maternal and child health services utilization in rural Ethiopia. European journal of epidemiology. 1993;9(5):511-6.	Data collected before 2002
13	Medhanyie A, Spigt M, Kifle Y, Schaay N, Sanders D, Blanco R, et al. The role of health extension workers in improving utilization of maternal health services in rural areas in Ethiopia: a cross sectional study. BMC Health Services Research. 2012;12:352.	This study focuses on the extent to which these trained community health workers have contributed to the improvement of utilization of maternal health services
14	Alemayehu M, Gebrehiwot TG, Medhanyie AA, Desta A, Alemu T, Abrha A, Godefy H. Utilization and factors associated with antenatal, delivery and postnatal Care	Due to outcome measurement

	Services in Tigray Region, Ethiopia: a community-based cross-sectional study. BMC Pregnancy Childbirth. 2020 Jun 1;20(1):334. doi: 10.1186/s12884-020-03031-6. PMID: 32487069; PMCID: PMC7268454.	
15	Mekonnen Y, Mekonnen A. Factors Influencing the Use of Maternal Healthcare Services in Ethiopia. Journal of health, population, and nutrition. 2003;21(4):374-82.	Old data (collected before 2002)
16	Nigatu D, Gebremariam A, Abera M, Setegn T, Deribe K. Factors associated with women's autonomy regarding maternal and child health care utilization in Bale Zone: a community based cross-sectional study. BMC Womens Health. 2014;14:79.	The dependent variable were women's autonomy and focus on knowledge of MCH
17	Tekelab T, Berhanu B. Factors Associated with Late Initiation of Antenatal Care among Pregnant Women Attending Antenatal Clinic at Public Health Centers in Kembata Tembaro Zone, Southern Ethiopia. Science, Technology and Arts Research Journal. 2014;3(1):108.	The aim of the study was late initiation of antenatal care
18	Gebresilassie B, Belete T, Tilahun W, Berhane B, Gebresilassie S. Timing of first antenatal care attendance and associated factors among pregnant women in public health institutions of Axum town, Tigray, Ethiopia, 2017: a mixed design study. BMC Pregnancy Childbirth. 2019 Sep 18;19(1):340. doi: 10.1186/s12884-019-2490-5. PMID: 31533657; PMCID: PMC6751589.	Focused on timing of first ANC
19	Wado YD, Afework MF, Hindin MJ. Unintended pregnancies and the use of maternal health services in Southwestern Ethiopia. BMC International Health & Human Rights. 2013;13:36.	The study participants are different from our inclusion criteria
20	Wakbulcho M, Möller B. Attitudes toward current pregnancy among women attending an antenatal clinic in Ethiopia. International Journal of Gynecology & Obstetrics. 1994;46(1):61-2.	The study examine attitude on pregnancy
21	Weldearegawi GG, Teklehaimanot BF,	Focused on determinants of late ANC follow-

	Gebru HT, Gebrezgi ZA, Tekola KB, Baraki MF. Determinants of late antenatal care follow up among pregnant women in Easter zone Tigray, Northern Ethiopia, 2018: unmatched case-control study. BMC Res Notes. 2019 Nov 19;12(1):752. doi: 10.1186/s13104-019-4789-8. PMID: 31744531; PMCID: PMC6862862.	up
22	Woldemicael G, Tenkorang EY. Women's autonomy and maternal health-seeking behavior in Ethiopia. Matern Child Health J. 2010;14(6):988-98.	The aim was on health seeking behaviour
23	Berhe KK , Welearegay HG , AberaGB , Kahsay HB, Kahsay AB. Assessment of Antenatal Care Utilization and its Associated Factors Among 15 to 49 Years of Age Women in Ayder Kebelle, Mekelle City 2012/2013; A Cross Sectional Study. American Journal of Advanced Drug Delivery	The result is not consistent. The finding in the abstract part indicated different from the main findings.
24	Gebretsadik A, Teshome M, Mekonnen M, Alemayehu A, Haji Y. Health Extension Workers Involvement in the Utilization of Focused Antenatal Care Service in Rural Sidama Zone, Southern Ethiopia: A Cross-Sectional Study. Health Serv Res Manag Epidemiol. 2019 Apr 4;6:2333392819835138. doi: 10.1177/2333392819835138. PMID: 30993149; PMCID: PMC6449814.	Outcome measurement, and reported inconsistent results.
25	Alemayehu T, Haidar J, Habte D. Utilization of antenatal care services among teenagers in Ethiopia: A cross sectional study. Ethiop. J. Health Dev. 2010;24(3):221-225	Data collected before 2002
26	Yilala S eta al. Assessment of late initiation of antenatal care and associated factors among antenatal care attendees in selected health centers of Addis Ababa, Ethiopia, 2015(Unpublished)	The outcome was late initiation of antenatal care.
27	Dulla D, Daka D, Wakgari N. Antenatal care utilization and its associated factors among pregnant women in Boricha District, southern Ethiopia. Divers Equal Health Care. 2017;14(2):76-84.	Reported outcome using odds ratio rather than AOR
28	Zegeye AM, Bitew BD, Koye DN. Prevalence and Determinants of Early Antenatal Care Visit among Pregnant Women Attending Antenatal Care in Debre Berhan Health Institutions, Central Ethiopia.	Focus on early antenatal care user.

	Afr J Reprod Health 2013; 17[4]: 130-136	
29	Woldemicael G. Do women with higher autonomy seek more maternal and child health-care? Evidence from Ethiopia and Eritrea	Mixed result from two countries
30	Umer A, Zinsstag J, Schelling E, Tschopp R, Hattendof J, Osman K, Yuya M, Ame A, Zemp E. Antenatal care and skilled delivery service utilisation in Somali pastoral communities of Eastern Ethiopia. Trop Med Int Health. 2020 Mar;25(3):328-337. doi: 10.1111/tmi.13346. Epub 2019 Dec 9. PMID: 31733130; PMCID: PMC7079025.	Lack of clear outcome definition
31	Muleta M, Gerrits T, Both R. Husbands' Roles in Prenatal Care in Addis Ababa. (unpublished thesis)	Use only qualitative methods and ANC user and non-user not mentioned clearly.
32	Gudayu T. Proportion and Factors Associated with late Antenatal Care Booking among Pregnant Mothers in Gondar Town, North West Ethiopia. Afr J Reprod Health 2015; 19[2]: 94-100	Describe only about late initiation of ANC
33	Suleman Hassen S, Mulatu Teshale B, Abate Adulo L. Identifying Factors Associated with Barriers in the Number of Antenatal Care Service Visits among Pregnant Women in Rural Parts of Ethiopia. ScientificWorldJournal. 2021 Oct 25;2021:7146452. doi: 10.1155/2021/7146452. PMID: 34733121; PMCID: PMC8560300.	Lack of clear outcome definition and inconsistent result
34	Sekata D. Modeling the Number of Antenatal Care Service Visits Among Pregnant Women in Rural Ethiopia: Zero Inflated and Hurdle Model Specifications. International Journal of Healthcare Sciences 2015. 3(1).	The result not clearly indicated and the analysis already done by Tarekegn SM et al, 2014
35	Mekonnen Y, Mekonnen A. Utilization of Maternal Health Care Services in Ethiopia. Calverton, Maryland, USA: ORC Macro.2002	The data collected before 2002
36	Terye ND. Multilevel Modeling of Utilization of Maternal Health Care Services in Ethiopia. Ethiopian e-journal for res and inn.2015;7(1)	Duplicate and statistical problem. The 2011 EDHS alredy analysed by Tarekegn SM et al, 2014
37	Tegegne TK, Chojenta C, Getachew T, Smith R, Loxton D. Antenatal care use in Ethiopia: a spatial and multilevel analysis. BMC Pregnancy Childbirth. 2019 Nov 1;19(1):399. doi: 10.1186/s12884-019-2550-x. PMID: 31675918; PMCID:	Due to outcome measurement

	PMC6825362.	
38	Aliy J, Hailemariam D. Determinants of equity in utilization of maternal health services in Butajira, Southern Ethiopia .Ethiop. J. Health Dev. 2012;26 Special Issue 1:265-270	The objective not specified and ANC user not indicated.
39	Endashaw T, Fantahun M. Assessment of late entry to antenatal care and its predictors among ANC attendees in Gambella region.June2010(Unpublished)	The study participants were ANC user.
40	Arefaynie M, Kefale B, Yalew M, Adane B, Dewau R, Damtie Y. Number of antenatal care utilization and associated factors among pregnant women in Ethiopia: zero-inflated Poisson regression of 2019 intermediate Ethiopian Demography Health Survey. Reprod Health. 2022 Feb 5;19(1):36. doi: 10.1186/s12978-022-01347-4. PMID: 35123503; PMCID: PMC8817592.	Due to outcome measurement
