# Supplementary material for: Predictors of Antenatal Care Service Utilization Among Women of Reproductive Age in Ethiopia: A Systematic Review and Meta-Analysis
Source: J Clin Med. 2025 Apr 7;14(7):2517. doi: 10.3390/jcm14072517 (PMC11989362; doi:10.3390/jcm14072517)
Supplement: Supplementary file 1 [file jcm-14-02517-s001.zip › Supplementary file 8.pdf]

Table 2: Description of essential features of studies included in the review, 2023

Author and year	Region	Study design	Data collection period	Study subject	Sample size	Response rate (%)	Predictors	Outcome	ANC use (%)
Kidist B et al, 2013	Oromia	CS	January to February 2012	Women who gave birth in the past three years	422	99.2	Maternal age and education status, income, media exposure, planned pregnancy, knowledge of ODS	MHC (ANC and SBA) Women who utilized at least one ANC service	87.1
Tesfaye G et al, 2018	Oromia	CS	June to August 2017	WRA, who gave birth in the three years before the survey	1294	100	Women's education status, previous utilization of ANC, residence, wealth index, husband's attitude about ANC	Women who utilized at least one ANC service	53.6
Jira C et al, 2005	Oromia	CS	Feb 1 to 20,2004	Pregnant women in their third trimester	307	100	Women's education and occupation status, perceived importance of ANC visit	Women who utilized at least one ANC service	90.6
Desalew ZA et al, 2014	Oromia	CS	June 2012	Women who gave at least one live	495	100	Women's age, education, family size, perceived	MHC (ANC and SBA) Women who	86.1

				birth in the 5 years			importance of ANC of services, residence	utilized at least one ANC service	
Bontu F, 2007	Oromia	CS	Jan-Feb,2006	Pregnant women in their third trimester	384	93.8	Women's education and occupation status, income, and religion	Women who utilized at least one ANC service	76.7
Abebaw GW et al, 2013	Amhara	Cohort study	Jan-Mar 2012	Women who had birthed in the last year preceding the survey	1730	96.4	Women's education, awareness on places to get skilled providers, previous pregnancy ANC use	MHC (ANC, SBA and PNC) Women who utilized at least one ANC service	32.3

Table 1: continued

Author and year	Region	Study design	Data collection period	Study subject	Sample size	Response rate (%)	Predictors	Outcome	ANC use (%)
Gebeyeu TN et al, 2015	Amhara	CS	Feb-Mar, 2013	WRA who had a birth in the past one year	317	96.8	Women's education and occupation status, income, plan of	Women who utilized at least one ANC service	55.7

							pregnancy, knowledge of ODS		
Yohannes AM et al, 2014	Tigray	Longitudinal cohort study	Sept-August, 2013	Pregnant and women who had given birth past 5 years	2361	98.2	Women's age, education, occupation, and residence	ANC and SBA Women who utilized at least one ANC service	76.5
Yalem T et al, 2013	Tigray	CS	Aug-Sep 2009	Women who had given birth in the five years	1,115	99	Age, marital status, education, parity, husbands Occupation	ANC and SBA Women who utilized at least one ANC service	54
Zeine A et al, 2010	SNNPR	CS	Janu-Feb,2009	Women who gave birth in the last five years	710	97.3	Women's age and education status, parity, planned pregnancy, knowledge of ODS	Women who utilized at least one ANC service	86.3
Abebaw AM et al, 2021	SNNPR	CS	Aug-Sept 30, 2018	WRA, who gave birth within six months of the survey	720	100	Education status of women and husbands, residence, wealth index	Women who utilized at least one ANC service	72.6
Zeleke D et al, 2015	SNNPR	CS	Jan 2014	Women who gave birth in the last year	634	98.2	Planned pregnancy, knowledge of ODS, education	MHC (ANC and SBA)	87.6

							status, occupation, women's autonomy	Women who utilized at least one ANC service	
Melese G et al,2016	SNNPR	CS	Jan-Feb,2015	Women who gave birth in the one year preceding the study	778	96.1	Residence, women and husband education, media exposure	Women who utilized at least one ANC service	71

Table 1: continued

Author and year	Region	Study design	Data collection period	Study subject	Sample size	Response rate (%)	Predictors	Outcome	ANC use (%)
Bahilu T et al, 2009	SNNPR	CS	April 2008	Women who had given birth in the past 12 months	651	96.3	Women's education, residence, planned pregnancy, knowledge of ODS	Women who utilized at least one ANC service	28. 5
Shegaw M et al, 2014	National	CS	Decem-June,2011	Women who gave at least one birth within the last 5 years	7908	100	Residence, education status, H.H. wealth status, husband education, the autonomy of	MHC (ANC and SBA) Women who utilized at least	33.9

							women, media exposure	one ANC service	
Muluwas A et al, 2015	Benishangul Gumuz	CS	May,2012	Women who gave birth in past five years	536	97.9	Place of residence, educational status, awareness on ANC service, distance	Women who utilized at least one ANC service	81.9
Gurmesa T G,2009	Benishangul Gumuz	CS	Janu-Feb 2007	Women gave birth at least one birth in the past 5 years	1060	97.9	Place of residence, women's education and occupation status, media exposure, wealth status	Women who utilized at least one ANC service	49.8
Kassahun T et al, 2019	Benishangul Gumuz	Regional CS	From Decem 2010 to June 2011	Mothers who had at least one child in the 5 years before the survey	916	100	Women's age and education, residence, religion, and ethnicity, wealth index	Women who utilized at least one ANC service	37.7
Tsegay B et al, 2021	Sidama	CS	Janu, 2019	Women who gave at least one birth in the last year preceding survey	692	98.67	Wealth index, education, media access, and plan pregnancy, information, and counseling about ANC	Skilled MHSU<br>Women who utilized at least one ANC service	69.1

N Regassa, 2011	Sidama	CS	2011	Women who had a child < 2 years	1094	100	Women's age, education and occupation status, media exposure, planned pregnancy	ANC and PNC Women who utilized at least one ANC service	77.4

Table 1: continued

Author and year	Region	Study design	Data collection period	Study subject	Sample size	Response rate (%)	Predictors	Outcome	ANC use (%)
Nejimu B et al, 2016	Afar	CS	Janu-Feb, 2015	Women who gave birth within 2 years prior to survey	788	100	Residence, occupation, income, source of information	Women who utilized at least one ANC service	42.4
Wubareg S et al, 2017	Somali	CS	June, 2011	Women who had childbirth in the past year	759	95.7	Maternal age, husband education, attitude, and parity	MHSU Women who received ANC at least once	66.2

ANC: Antenatal care; CS: Cross-sectional; MHSU: Maternal health service utilization; ODS: Obstetric danger signs; PNC: Postnatal care; SBA: Skilled birth attendant; SNNPR: South Nations Nationalities and Peoples Regional State
