# Supplementary material for: Predictors of Antenatal Care Service Utilization Among Women of Reproductive Age in Ethiopia: A Systematic Review and Meta-Analysis
Source: J Clin Med. 2025 Apr 7;14(7):2517. doi: 10.3390/jcm14072517 (PMC11989362; doi:10.3390/jcm14072517)
Supplement: Supplementary file 1 [file jcm-14-02517-s001.zip › Supplementary file 9.pdf]

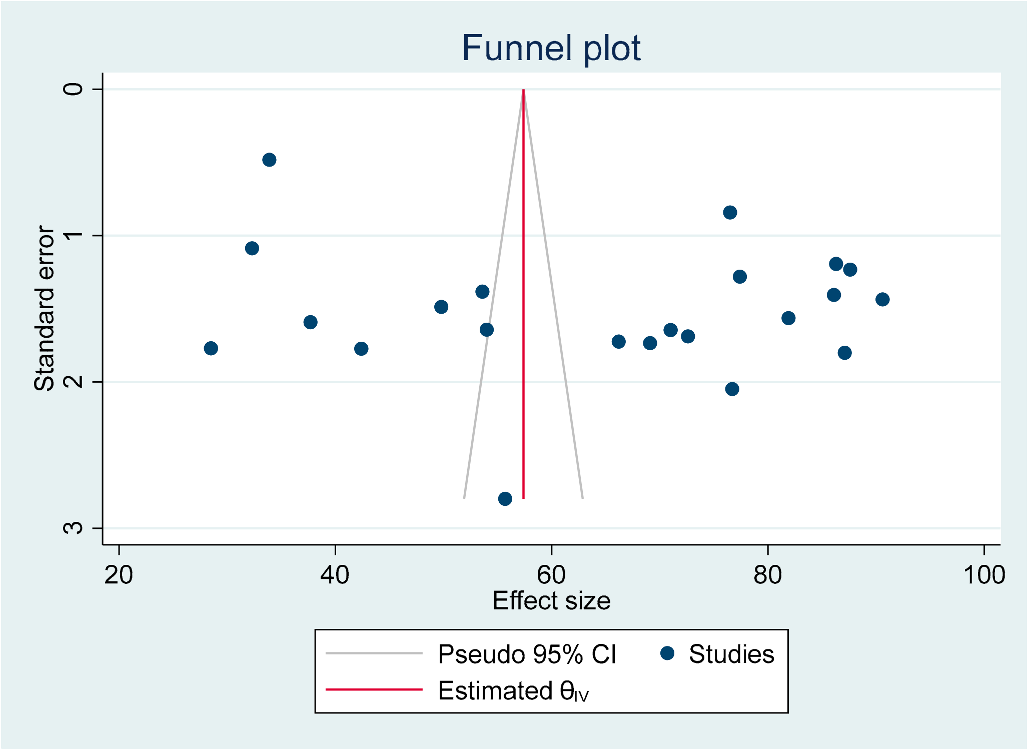

Figure 1: The funnel plot to check publication bias of overall pooled ANC service utilization among women of reproductive age in Ethiopia, 2022.

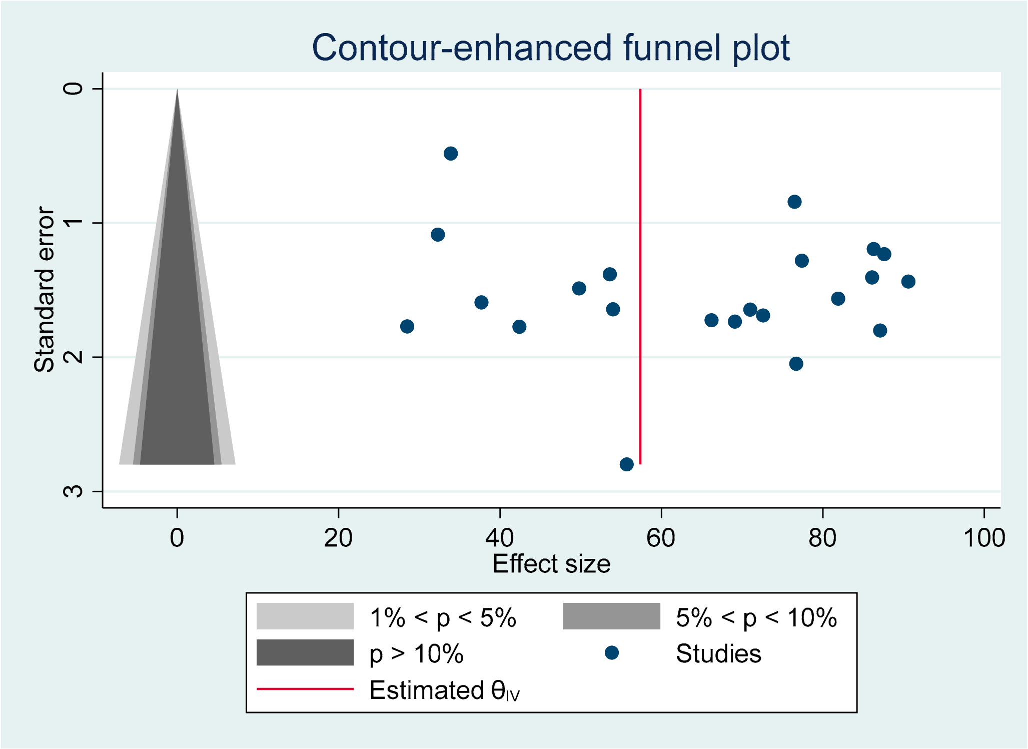

Figure 2: The contour-enhanced funnel plot to check publication bias of overall pooled ANC service utilization among women of reproductive age in Ethiopia, 2022.
